# CD8α Structural Domains Enhance GUCY2C CAR-T Cell Efficacy

**DOI:** 10.1080/15384047.2024.2398801

**Published:** 2024-09-24

**Authors:** Trevor R. Baybutt, Ariana A. Entezari, Adi Caspi, Ross E. Staudt, Robert D. Carlson, Scott A. Waldman, Adam E. Snook

**Affiliations:** a Department of Pharmacology, Physiology, and Cancer Biology, Sidney Kimmel Medical College, Thomas Jefferson University, Philadelphia, PA, USA; b Sidney Kimmel Comprehensive Cancer Center, Jefferson Health, Philadelphia, PA, USA; c Department of Microbiology & Immunology, Sidney Kimmel Medical College, Thomas Jefferson University, Philadelphia, PA, USA

**Keywords:** Chimeric antigen receptor, CAR-T cell therapy, GUCY2C, colorectal cancer

## Abstract

Despite success in treating some hematological malignancies, CAR-T cells have not yet produced similar outcomes in solid tumors due, in part, to the tumor microenvironment, poor persistence, and a paucity of suitable target antigens. Importantly, the impact of the CAR components on these challenges remains focused on the intracellular signaling and antigen-binding domains. In contrast, the flexible hinge and transmembrane domains have been commoditized and are the least studied components of the CAR. Here, we compared the hinge and transmembrane domains derived from either the CD8ɑ or CD28 molecule in identical GUCY2C-targeted third-generation designs for colorectal cancer. While these structural domains do not contribute to differences in antigen-independent contexts, such as CAR expression and differentiation and exhaustion phenotypes, the CD8ɑ structural domain CAR has a greater affinity for GUCY2C. This results in increased production of inflammatory cytokines and granzyme B, improved cytolytic effector function with low antigen-expressing tumor cells, and robust anti-tumor efficacy *in vivo* compared with the CD28 structural domain CAR. This suggests that CD8α structural domains should be considered in the design of all CARs for the generation of high-affinity CARs and optimally effective CAR-T cells in solid tumor immunotherapy.

## Introduction

In the seven years since chimeric antigen receptor (CAR) T-cell therapy was approved by the FDA for the treatment of hematological malignancies, there have been no approvals for any CAR-T cell therapy directed towards a solid tumor antigen. The consensus within the field has identified three major obstacles that contribute to the inability of CAR-T cells to successfully eradicate solid tumor lesions: (1) trafficking of the CAR-T cells to, and ingress of T cells within, the solid tumor space, (2) the persistence and concomitant functions of CAR-T cells within the suppressive tumor microenvironment, and (3) identifying a suitable tumor antigen that promotes tumor eradication while sparing the surrounding healthy tissue.^1,2^

In the context of colorectal cancer (CRC), it has been demonstrated clinically, both with checkpoint blocking therapy and adoptively transferred autologous T cells, that metastatic lesions arising from the colon can be controlled by cytolytic T cells.^3–5^ However, these therapeutic options are limited by specific disease states as well as loss of the antigen-presenting major histocompatibility complex (MHC) molecule in tumor cells. Moreover, T-cell therapy utilizing the T-cell receptor (TCR) as the tumor-antigen targeting modality requires a degree of personalization above the requirements of CAR-T cells, which use an antibody-derived targeting modality against a native cell surface molecule for broad application to the patient population.

Our laboratory identified guanylyl cyclase C (GUCY2C) as a mucosal antigen within the intestinal epithelium that can be targeted using immunotherapies.^6,7^ GUCY2C was identified as a particulate cyclase and molecularly characterized as the receptor for the *E. coli* heat-stable enterotoxin ST, responsible for diarrheal disease.^8–10^ The luminal restriction of the extracellular domain of this type I transmembrane receptor can be leveraged therapeutically, as GUCY2C-expressing metastatic lesions originating from primary colonic adenocarcinoma are no longer topologically sequestered within the luminal space, making the extracellular domain a viable antigenic target for systemically delivered therapies.^11^ While the cyclase activity of GUCY2C is silenced in CRC, receptor expression is maintained in > 95% of cases.^12^ Immunotherapeutic modalities including vaccines, antibody-drug conjugates, bi-specific T-cell engagers, and CAR-T cells have demonstrated efficacy in controlling or eliminating GUCY2C-expressing metastatic lesions in animal models.^13–19^ GUCY2C has also proven to be a safe target in a mouse model of a GUCY2C-directed syngeneic CAR-T cell therapy, and there was no toxicity attributable to the targeted therapy.^20^ Moreover, in a clinical trial of a GUCY2C-targeted adenoviral cancer vaccine there were no adverse events reported greater than grade 1, suggesting that T cells directed toward GUCY2C do not promote intestinal colitis.^21^

The general design of CARs for expression by T cells has remained relatively homogenous since their inception, consisting of (1) an antigen-targeting domain, typically derived from an antibody and organized into a single molecular framework called a single chain variable fragment (scFv), (2) structural domains consisting of a flexible molecular hinge and transmembrane domain (HTM), and (3) intracellular signaling domains (ICDs) derived from T-cell proteins localized to the immunological synapse. In CAR design, the largest variability is with the scFv, due to the array of different antigenic targets, and then the ICDs, which have predominantly consisted of CD3ζ, CD28, and 4-1BB. The least modified region, and the least well understood in terms of the role these domains play in CAR efficacy, are the structural domains. The most prominently used domains come from either CD8ɑ or CD28; however, other domains derived from IgG4 have been utilized. The CARs that led to the first two FDA-approved therapies targeting CD19 using the same scFv were combined with either the CD8ɑ hinge and transmembrane domains plus the 4-1BB and CD3ζ ICDs (Tisagenlecleucel, Kymriah, Novartis AG) or the CD28 hinge and transmembrane domains with CD28 and CD3ζ ICDs (Axicabtagene Ciloleucel, Yescarta, Kite Pharma, Inc.), referred to as either a BBζ or a 28ζ design based upon the ICD configuration.^22,23^

Having identified a human GUCY2C-specific scFv and performed mouse studies using a CD28, 4-1BB, and CD3ζ (28BBζ) ICD design, we sought to determine if the previously overlooked structural domains, derived from either CD8ɑ or CD28, had an impact on CAR efficacy and CAR-T cell phenotypes. We designed two CARs, which we call the CD8HTM (represented by all blue data) or the CD28HTM (represented by all red data) CAR, that differed only in the structural domains, and evaluated the *in vitro* and *in vivo* performance of these two receptors for the treatment of metastatic CRC.

## Results

### CAR-T cell manufacturing parameters are not impacted by structural domains

**Figure 1.**
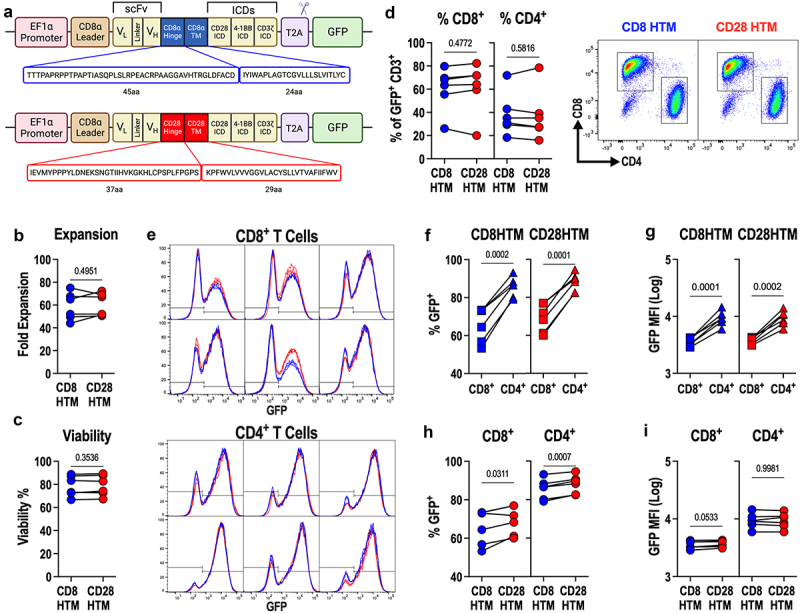
CD8HTM and CD28HTM GUCY2C CAR-T Production. (a) designs of CD8HTM and CD28HTM CARs. (b-i) CD8HTM and CD28HTM CAR constructs used were from 14-day manufacturing of CAR-T cells from *n* = 6 donors and matched comparisons between CARs. (b-c) expansion and viability of CD8HTM and CD28HTM CAR-T cell products. (d) CD8^+^ and CD4^+^ populations among CAR-transduced T cells. Flow cytometry plots shown. (e) Transduction efficiency (% GFP^+^) and construct expression (GFP MFI) for all 6 donors. (f-g) comparison of transduction efficiency (f) and construct expression (g) between CD8^+^ and CD4^+^ T cells for each CAR. (h-i) comparison of transduction efficiency (h) and construct expression (i) between CD8HTM and CD28HTM CARs for CD8^+^ and CD4^+^ T cells. All statistical comparisons are paired T-tests; symbols connected with a line represent a matched donor.

To assess the role that the hinge and transmembrane domains have on CAR-T cell efficacy, we employed a third-generation design which we have previously demonstrated exhibits robust anti-tumor efficacy and safety in mouse models.^19,20^ The CAR expression cassette consists of an EF1ɑ promoter and the following human sequences: CD8ɑ leader sequence, anti-GUCY2C (Clone 5F9) single chain variable fragment (scFv) with a (G4S)_4_ linker, hinge and transmembrane domains (HTM) from CD8ɑ (CD8HTM) or CD28 (CD28HTM), the CD28 ICD, 4-1BB ICD, and CD3ζ ICD, followed by the virally-derived T2A self-cleavable peptide and green fluorescent protein (GFP) reporter (Figure 1a). CAR-T cells were expanded ex vivo for 12 days using G-Rex plates before cryopreservation. At the conclusion of this culture period, there was no difference in the expansion of the CAR-T cell products between the CD8HTM and CD28HTM CARs (Figure 1b). We also observed no impact of the structural domains on T-cell viability (Figure 1c). The GFP reporter was used as a marker of lentivirus-transduced T cells in flow cytometric analyses. Of GFP^+^ CD3^+^ T cells, the structural domains did not impact the expansion of the CD8^+^ or CD4^+^ co-receptor subsets (Figure 1d). While the majority of donors had a greater percentage of CD8^+^ T cells after expansion, one donor exhibited a greater expansion of the CD4^+^ population relative to the CD8^+^ population; however, the other characteristics of the T cells from this donor did not differ from the other donors. For each CAR construct, we observed a statistically significant difference in the transduction of CD8^+^ and CD4^+^ T cells, with the CD4^+^ T cells having a higher percentage of GFP^+^ cells (Figure 1e-f) and higher levels of construct expression (GFP MFI; Figure 1g) in both the CD8HTM and CD28HTM CAR-T cells. This significant difference in the transduction efficiency between CD8^+^ and CD4^+^ T cells has been reported previously in CAR-T cell studies using VSV-G pseudotyped lentivirus^24,25^ and necessitates that these two populations be examined separately. Moreover, the transduction efficiency of CD8^+^ T cells and CD4^+^ T cells is slightly higher with the CD28HTM than CD8HTM CAR (Figure 1h); however, the expression of each construct (GFP MFI) was equivalent between the CD8HTM and CD28HTM CARs (Figure 1i).

### Structural domains do not affect antigen-independent T-cell phenotypes

It has been demonstrated that the quality of infused T-cell products has a significant impact on patient outcomes.^26^ Less differentiated memory phenotypes, such as naïve-like/stem cell memory-like and central memory phenotypes, are favored over more differentiated effector memory and effector T cells.^27^ Similarly, T cells expressing high levels of exhaustion markers at baseline also lead to poor therapeutic outcomes.^28^ Although components of the CAR such as the scFv and ICDs can contribute to memory phenotypes and exhaustion, it is unclear what role the structural domains have in the outcome of T-cell phenotypes. It should be noted that these phenotypes occur independently of target-antigen exposure. Although T cells are activated using anti-CD3/CD28/CD2 antibody agonism, antigen-dependent signaling mediated through the CAR has not yet occurred for these CAR T cells. Differences in memory T-cell phenotypes influenced by the CAR have been shown to be mediated by differences in intracellular signaling domains.^29^ High tonic signaling through the CAR, leading to increased T-cell exhaustion, has been shown to be mediated by intrinsic qualities of the scFv.^30,31^ Given that the CD8HTM and CD28HTM CARs have identical scFvs, ICDs, and surface-level expression, we hypothesized that these antigen-independent T-cell phenotypes would be similar between the two CARs.

**Figure 2.**
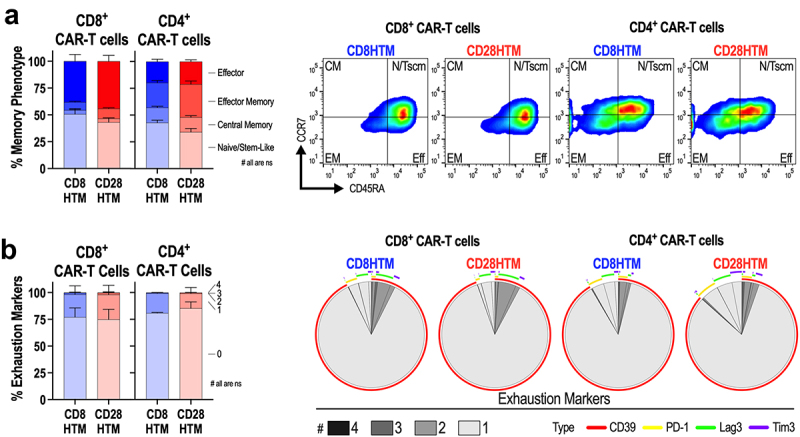
CD8HTM and CD28HTM CAR-T products lack exhaustion and possess memory phenotypes. (a) effector, effector memory, central memory, and naïve/stem cell memory-like phenotypes among matched CD8HTM and CD28HTM CAR-T cells produced from *n* = 3 donors. Representative flow cytometry plots are shown. (b) prevalence of 0 to 4 exhaustion markers expressed among matched CD8HTM and CD28HTM CAR-T cells produced from 3 donors. SPICE plots demonstrating the average number and type of exhaustion marker expression. CD8HTM and CD28HTM memory (a) and exhaustion (b) phenotypes were not statistically different. Statistical analyses used two-way ANOVA adjusted for multiple comparisons. Error bars reflect the standard error of the mean (SEM).

The four major memory cell populations were defined using the markers CD45RA and CCR7.^32–34^ In comparing the four major memory cell phenotypes: naïve/stem cell memory-like (N/Tscm), central memory (CM), effector memory (EM), and effector (Eff) cells, we observe no statistically significant difference between the CD8HTM and CD28HTM CAR-T cells in the CD8^+^ or CD4^+^ T-cell populations (Figure 2a). In terms of exhaustion marker expression, no statistically significant difference was observed between the CD8HTM and CD28HTM CAR-T cells as measured by the number of exhaustion markers expressed per cell in both the CD8^+^ and CD4^+^ T-cell populations (Figure 2b). The majority of cells do not express any exhaustion markers (~75-85% negative across CARs and T-cell subsets). Of the cells producing at least one exhaustion marker, that marker, as evidenced by the Simplified Presentation of Incredibly Complex Evaluations (SPICE) plots, is CD39 (Figure 2b). When total CD39 expression is evaluated between the CD8HTM and CD28HTM CAR-T cells, there is no statistically significant difference in either the CD8^+^ (*p* = .1924) or CD4^+^ (*p* = .2042) T-cell populations.

### T cells display similar amounts of CD8HTM and CD28HTM CARs

**Figure 3.**
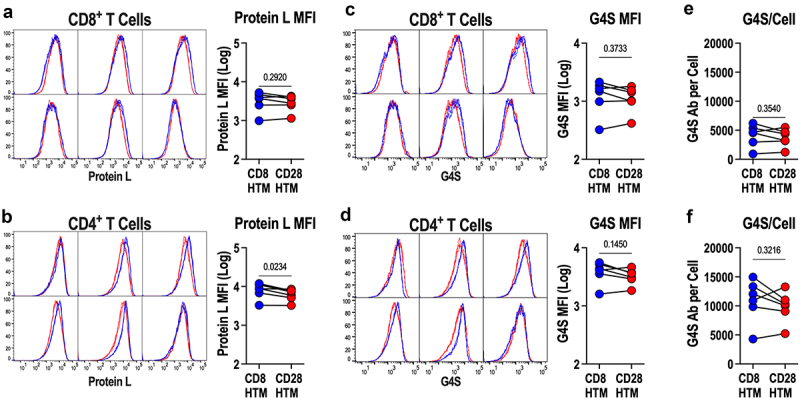
CD8HTM and CD28HTM CAR expression. (a-b) surface CD8HTM and CD28HTM CAR expression was examined with protein L among CD8^+^ (a) and CD4^+^ (b) CAR-T cells produced from *n* = 6 donors. (c-d) surface CD8HTM and CD28HTM CAR expression was examined with anti-G4S antibody among CD8^+^ (c) and CD4^+^ (d) CAR-T cells produced from 6 donors. (e-f) bound anti-G4S antibodies/cell in c-d were quantified among CD8^+^ (e) and CD4^+^ (f) CAR-T cells. Flow cytometry plots in a-d indicate matched CD8HTM and CD28HTM CAR expression for each donor. All statistical comparisons are paired T-tests; symbols connected with a line represent a matched donor.

Protein L (*Peptostreptococcus magnus*) was used to measure CAR surface levels (Figure 3a-b). Protein L binds to the kappa light chain of antibodies, allowing for an antigen-independent method for detecting CAR by binding to the light chain of the scFv, revealing comparable levels of CD8HTM and CD28HTM CAR among CD8^+^ (Figure 3a) and CD4^+^ (Figure 3b) T cells. The two antibody variable domains that comprise the scFv are connected by a flexible linker composed of four glycine residues and one serine. This peptide subunit is then concatenated four times to produce the (G4S)_4_ flexible linker. Similar to Protein L, an antibody directed against this peptide linker can be used to identify CAR molecules on the surface of the T cell, independent of antigen binding, through linker binding (Figure 3c-f). The CD8HTM and CD28HTM CAR designs are represented in equivalent amounts on the surface of CD8^+^ (Figure 3c) and CD4^+^ (Figure 3d) T cells, determined by G4S antibody MFI. Using a PE-labeled anti-G4S antibody, we were able to quantify the number of CAR molecules on the T-cell surface, revealing similar levels of CD8HTM and CD28HTM CARs in CD8^+^ (Figure 3e) and CD4^+^ (Figure 3f) T cells. Given that CD4^+^ T cells have a higher percentage of GFP positive cells with higher MFIs compared with matched CD8^+^ T cells (Figure 1), we expect that, given the stoichiometric equivalency of proteins produced upstream and downstream of the T2A cleavage site, CD4^+^ T cells would have more CAR on their surface, which is indeed what we observed. CAR molecules per T cell were quantified with the mean molecules/cell for CD8^+^ T cells being 3906 and for the CD4^+^ T cells being 10,365 (*p* < .0001), regardless of CAR design.

### Antigen binding is improved using the CD8 hinge and transmembrane domains

**Figure 4.**
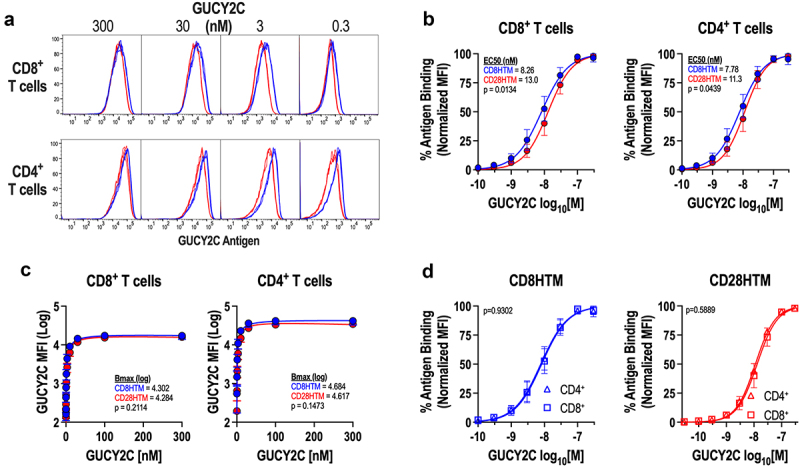
Enhanced CD8HTM CAR affinity compared to CD28HTM CAR. (a-d) purified recombinant GUCY2C extracellular domain was incubated with CD8HTM and CD28HTM CAR-T cells produced from *n* = 3 donors at varying concentrations (0-300 nM). Bound GUCY2C was detected with a fluorescent secondary antibody and cells were analyzed by flow cytometry. (a) Representative flow cytometry plots comparing CD8HTM and CD28HTM CARs. (b) affinity and EC50 determination for CD8HTM and CD28HTM CARs among CD8^+^ and CD4^+^ CAR-T cells. (c) maximum binding (bmax) determination for CD8HTM and CD28HTM CARs among CD8^+^ and CD4^+^ CAR-T cells. (d) comparison of CAR affinities between CD8^+^ and CD4^+^ T cells for each CAR design. Non-linear regression analysis was performed in b-d with p-value testing for matching curves.

With the assessment of CAR surface expression performed using labeling methods independent of target antigen binding, we next wanted to ask if the structural domains of the CD8HTM and CD28HTM CARs affect the affinity of the scFv for its antigenic target, GUCY2C (Figure 4a-b). CD8HTM and CD28HTM CAR-T cells were incubated with a wide range of concentrations of the soluble extracellular domain of GUCY2C to establish a binding curve. GUCY2C binding to the CAR was measured using flow cytometry. MFI values were normalized within each CAR to generate relative binding affinities for comparisons. This revealed a difference in affinity for GUCY2C between the two CARs, with the CD8HTM CAR having a higher affinity for antigen (Figure 4a-b). Non-linear regression modeling revealed the CD8HTM CAR to have an EC50 of 7.78–8.26 nM, whereas the CD28HTM CAR had an EC50 of 11.3–13.0 nM in CD8^+^ and CD4^+^ T cells (Figure 4a-b). While there is a significant difference in antigen affinity (Figure 4b), plotting the dose with actual (non-normalized) binding reveals a similar total receptor occupancy (Bmax), when saturated concentrations of ligand are used (Figure 4c). This corroborates our findings using antigen-independent detection methods (Figure 3), demonstrating that T cells are expressing equivalent amounts of CAR molecules on the T-cell surface. We next determined if there were also affinity differences within a single CAR design between the CD8^+^ and CD4^+^ T-cell populations (Figure 4d). As expected, non-linear regression modeling revealed that there is no difference in CD8HTM or CD28HTM affinity between CD8^+^ and CD4^+^ T-cell populations (Figure 4d). Therefore, it is the structural domains themselves that contribute to the difference in antigen affinity (Figure 4a-b), and not the quantity of CAR receptor or intrinsic differences inherent to T-cell co-receptor subsets.

### Antigen exposure results in a higher level of effector cytokine production in CD8HTM CAR-T cells

**Figure 5.**
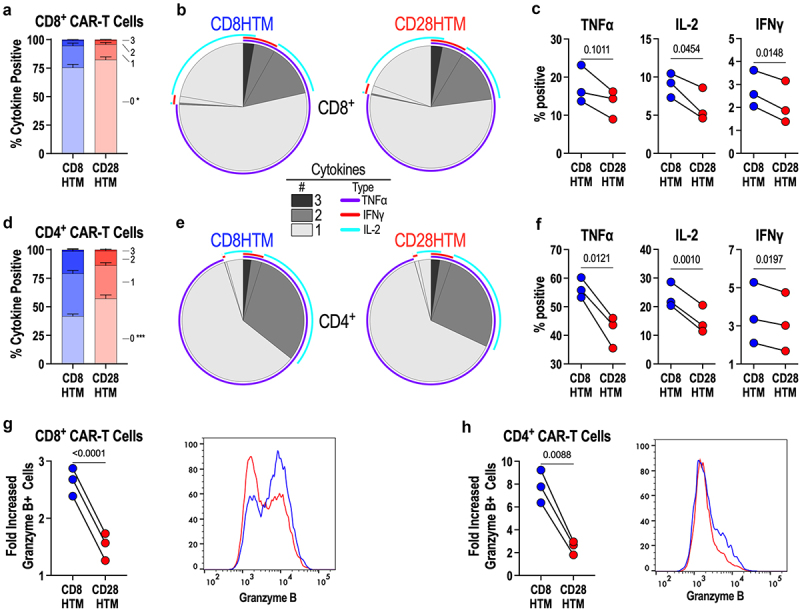
Enhanced cytokine production and cytolytic potential by CD8HTM CAR compared to CD28HTM CAR. (a-h) CD8HTM and CD28HTM CAR-T cells produced from *n* = 3 donors were stimulated with plate-bound recombinant GUCY2C extracellular domain protein, and intracellular cytokines (a-f), and granzyme B (g-h) were quantified by flow cytometry. (a,d) comparison of CD8^+^ T cells (a) and CD4^+^ T cells (d) producing 0-3 cytokines between CD8HTM and CD28HTM CARs. * *p* = .048; *** *p* = .0005 (b) SPICE plots demonstrating the average number and type of cytokine expression by CD8HTM and CD28HTM CAR-T cells among CD8^+^ (b) and CD4^+^ (e) CAR-T cells. Individual cytokine expression comparison between CD8HTM and CD28HTM CAR-T cells among CD8^+^ (c) and CD4^+^ (f) CAR-T cells. Granzyme B comparison between CD8HTM and CD28HTM CAR-T cells among CD8^+^ (g) and CD4^+^ (h) CAR-T cells. Representative flow cytometry plots are shown. Statistical analyses for a and d were performed using two-way ANOVA adjusted for multiple comparisons. Error bars reflect the SEM. Statistical comparisons for c, f, g, and h are paired T-tests; symbols connected with a line represent a matched donor.

Although target cell lysis is the primary effector function of CAR-T cells, the production of inflammatory effector molecules is critical for the bolstering and sustainability of the anti-tumor response.^35^ To assess the ability of these two CARs to produce polyfunctional inflammatory cytokine responses upon antigen stimulation, intracellular cytokine production was measured after six hours of plate-bound antigen exposure, followed by intracellular cytokine staining, and flow cytometry. Overall, the production of inflammatory cytokines is not robust with this specific CAR; however, we observed a statistically significant improvement in the ability of the CD8HTM CAR to produce cytokines after stimulation as compared with the CD28HTM CAR (Figure 5a-b). However, the fractions of the population representing the production of one, two, or three inflammatory cytokines are equivalent between the two CARs (Figure 5a-b). Of the CD8^+^ CAR-T cells that produce cytokines upon antigen encounter, we used SPICE plots to demonstrate the similarities in polyfunctionality, in not only the number of cytokines produced within a specific cell but also the specific combinations of TNFɑ, IFNɣ, and IL-2 that contribute to this polyfunctional cytokine response (Figure 5b). In comparing the cytokine production within the total CD8^+^ T-cell population, CD8HTM and CD28HTM CAR-T cells produce similar amounts of TNFɑ (Figure 5c). However, there are more IL-2 and IFNɣ producing CD8HTM CAR-T cells than the CD28HTM CAR-T cells (Figure 5c).

The CD4^+^ CAR-T cells, with more CAR molecules expressed on the T-cell surface, have an expectedly more robust cytokine response compared with the CD8^+^ T cells. However, like the CD8^+^ T-cell population, we observe the same increase in CD8HTM CAR-T cells able to produce ≥ 1 cytokine compared to the CD28HTM CAR-T cells (Figure 5d). Like CD8^+^ T cells, the degree of polyfunctionality between the two CARs is equivalent (Figure 5d-e). Unlike the CD8^+^ T-cell population, there was a statistically significant increase in the percentage of the CD4^+^ T-cell population producing each of the three inflammatory cytokines that were evaluated (Figure 5f). In addition to the inflammatory cytokines, we also observed that CD8^+^ CAR-T cells expressing the CD8HTM produce significantly more of the cytolytic effector molecule granzyme B after antigen stimulation than those expressing the CD28HTM CAR (Figure 5g). Interestingly, CD4^+^ CAR-T cells are also capable of executing cytolytic activity, and we see a statistically significant increase in the CD4^+^ CD8HTM CAR-T producing granzyme B after antigen exposure (Figure 5h). Although the granzyme B production is expectedly not as robust as in the CD8^+^ T cells, GUCY2C exposure results in significantly more granzyme B^+^ CD8HTM CAR-T cells than CD28HTM CAR-T cells. It should be noted that both CD8HTM and CD28HTM CAR-T cells have equivalent granzyme B^+^ percentages of the population with control peptide stimulation.

### CD8HTM CAR demonstrates superior in vitro killing when target antigen is lowly expressed

**Figure 6.**
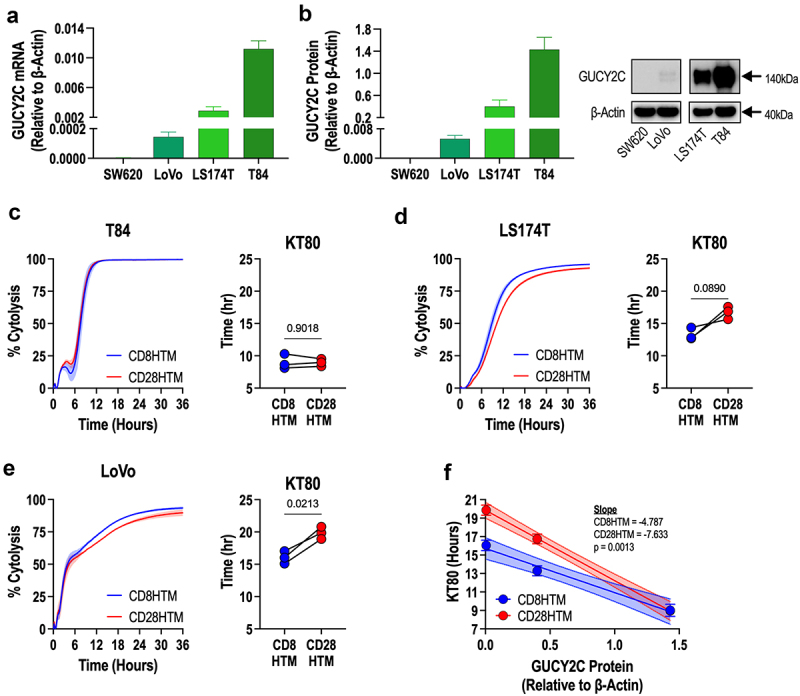
Enhanced cytolysis at decreasing antigen densities by CD8HTM CAR compared to CD28HTM CAR. (a-b) GUCY2C mRNA (a) and protein (b) were quantified for colorectal cancer cells spanning from undetectable to high expression. (c-e) cytolysis kinetics and time-to-80%-killing (KT80) comparisons between CD8HTM and CD28HTM CARs from *n* = 3 donors using T84 (c), LS174T (d), and LoVo (e) cells. (f) Correlation of KT80 and GUCY2C protein levels from *n* = 3 donor CAR-T cells. Shaded regions surrounding the cytotoxicity curves (c,d,e) represent the SEM. Statistical comparisons for the KT80 graphs (c,d,e) are paired T-tests; symbols connected with a line represent a matched donor. Error bars in (f) represent the SEM; the 95% confidence interval is represented by the shaded region.

To assess if the higher antigen affinity observed in CD8HTM CARs confers a functional benefit *in vitro*, we identified metastatic CRC cell lines with a wide range of GUCY2C expression to test with *in vitro* killing assays: LoVo (low-expression), LS174T (medium expression), and T84 (high-expression) using RT-PCR analysis (Figure 6a). Western blot analysis revealed a similar pattern of expression between GUCY2C mRNA and protein levels (Figure 6b). GUCY2C protein appears as a doublet with the two bands between 130 to 150 kDa.

We hypothesized that the differences in affinity for GUCY2C would be revealed inversely to the antigen density. In the high GUCY2C expressing T84 cells, there is no difference in the ability of the CD8HTM and CD28HTM CARs to kill *in vitro* as demonstrated using the real-time cell killing xCELLigence assay (Figure 6c). Both CARs are able to kill the total T84 population within 12 hours of adding the CAR-T cells, with equivalent time to 80% killing of the target cell population (KT80) of ~9 hours for each CAR (Figure 6c). In the medium GUCY2C expressing cell line (LS174T), there was an expected slowing of the tumor killing kinetics (Figure 6d). Moreover, there is a noticeable divergence in the curves where the CD8HTM CAR-T cells appear to have more complete killing of the target cells than the CD28HTM CAR-T cells (Figure 6d). Although the CD8HTM CAR was 3 hours faster in achieving the KT80, the difference was not statistically significant (*p* = .0890, Figure 6d). This suggested that LS174T cells are on the threshold of antigen density, where lower amounts of target antigen will result in significant effects. Cytolysis of the low-expressing LoVo cells is slower than T84 and LS174T and reveals a significant divergence between CD8HTM and CD28HTM CARs (Figure 6e). Importantly, there is a strong correlation between antigen density (GUCY2C protein levels) and CAR efficacy (KT80; Figure 6f). Moreover, linear regression demonstrates an increasing difference between CD8HTM and CD28HTM CARs at decreasing antigen densities (Figure 6f).

### CD8HTM structural domain CARs exhibit robust and durable anti-tumor responses

We have demonstrated that functional differences between the two CARs are revealed when the CAR is engaging antigen (affinity, cytokine production, granzyme B induction, and cytolysis). *In vitro*, this sensitivity is revealed in the context of low antigen densities; however, *in vitro* killing is not a rate limiting step for most CAR-T cells, as even the very low antigen-expressing LoVo cells were successfully killed by both CARs. CAR-T cell efficacy is the culmination of cumulative T-cell responses that inform this living drug to functionally eradicate tumors. To assess whether there are inherent differences between these two CAR designs in an *in vivo* tumor model, we decided to evaluate these two CAR T cells in a system where antigen was not a limiting factor of efficacy, purposefully biasing the system toward parity. We selected the T84 cell line, with the highest expression level of the antigen, and where, *in vitro*, there was no difference in the ability of these CAR-T cells to completely eradicate the tumor cells.

**Figure 7.**
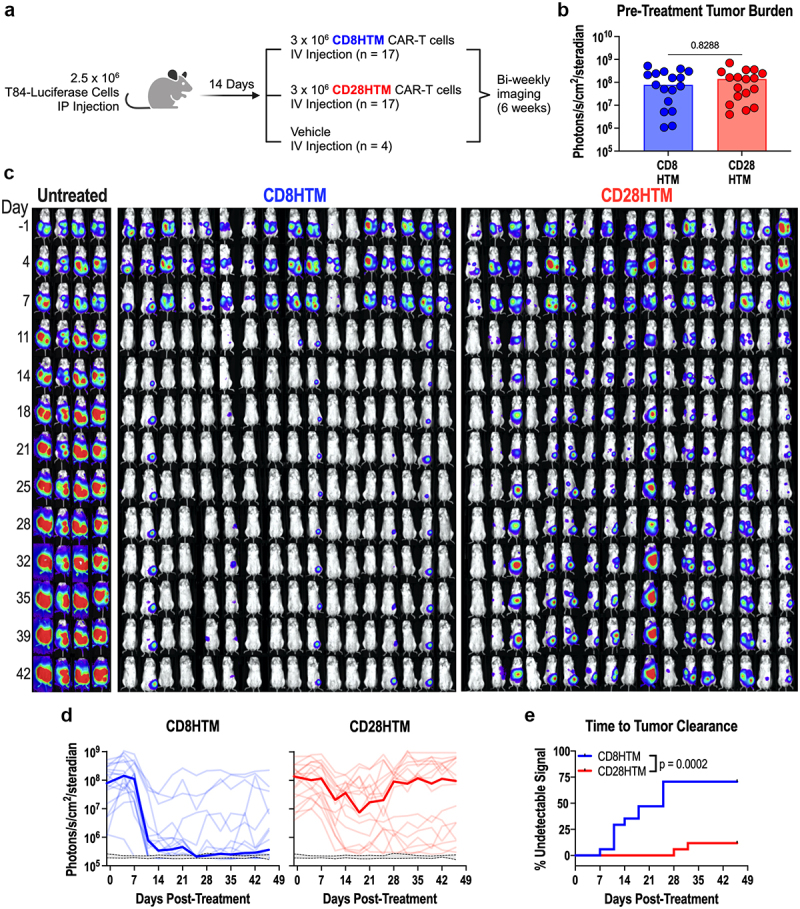
Enhanced antitumor efficacy of CD8HTM CAR compared to CD28HTM CAR. (a) experimental design (created with BioRender.com/w13b661). (b) Tumor burden comparison between CD8HTM and CD28HTM groups one day before treatment. (c) Longitudinal bioluminescence images. (d) Median (bold) and individual tumor burden comparison between CD8HTM and CD28HTM CAR-T cells. Grey range with dashed lines indicates the 95% confidence interval of baseline daily luminescence in tumor-free mice. (e) Time to tumor clearance comparison between CD8HTM and CD28HTM CAR-T cells determined by the day an animal’s signal reached that of tumor-free mice in d. An unpaired T-test was performed in b. Each dot represents a single animal. A Kaplan-Meier curve was used in e with the Log-rank (Mantel-Cox) test to determine the p-value.

T84 cells were engineered to express Click Beetle Red luciferase with a T2A self-cleavable peptide sequence followed by the mCherry fluorescent reporter (used for selection). NOD-*scid*-gamma MHC Class I/II double knock-out (NSG-MHC I/II DKO) mice were injected with 2.5 million T84 cells into the intraperitoneal cavity, modeling peritoneal metastases.^36^ Fourteen days after tumor administration, animals received 3 million CD8HTM or CD28HTM CAR-T cells, and 4 animals received vehicle. An additional 5 mice were injected with a vehicle on the day of the tumor implant to establish the daily luminescence level of tumor-free animals. All animals were imaged bi-weekly to assess tumor burden by luminescence (Figure 7a). Tumor-challenged animals with luminescence signals falling within the range (per imaging day) of these tumor-free mice would be deemed to have an undetectable signal.

Animals were imaged on the day prior to CAR-T cell treatment and rank-ordered by luminescence intensity. The four animals surrounding the median of the list were assigned to the untreated group, so that they would have comparable levels of tumor burden. The remaining animals were assigned to groups using a block randomization design, so that both CAR-T treatment groups would have representative animals across the range of tumor burdens. Comparing the luminescence signal one day before treatment, both groups were evenly distributed (Figure 7b).

*In vivo* bioluminescence imaging was performed bi-weekly, revealing a robust reduction in the luminescence signal 11 days after CAR-T cell treatment (Figure 7c-d). As expected, the signal from untreated animals increased over the course of the experiment. Importantly, the CD8HTM CAR-T cells produced robust tumor elimination compared to the CD28HTM CAR-T cells (Figure 7c-d). Using the tumor-free animals as the baseline luminescence of a mouse, we next wanted to ask about the rate of tumor clearance between the CD8HTM and CD28HTM CAR-T cells. A Kaplan-Meier curve was employed to demonstrate the first day at which an animal had a luminescence signal comparable to the tumor-free animals (Figure 7e). There was a 21-day period between when the first CD8HTM treated mouse became undetectable (Day 7) versus the first CD28HTM treated mouse (Day 28). In total, tumors were cleared in 70.6% of the CD8HTM-treated group, while only 11.8% of the CD28HTM-treated animals were cleared (Figure 7e). These data demonstrate that the hinge and transmembrane domains derived from the CD8ɑ molecule have superior antitumor efficacy compared with the CD28HTM design for this anti-GUCY2C CAR.

## Discussion

Optimal CAR design is paramount to efficacy, yet empirical comparisons are often limited to comparisons of scFv or ICD configurations, with limited comparison of structural domains thought to have modest impacts on CAR function. Here, we revealed that a CD8α hinge and transmembrane provides superior avidity, *in vitro* effector function, and *in vivo* antitumor efficacy targeting the CRC antigen GUCY2C in the context of a “3^rd^ generation” 28BBζ CAR. It has been demonstrated that independently, the co-stimulatory domains contribute different functional characteristics to CAR-T cells, with the CD28 ICD promoting faster cytolytic kinetics while the 4-1BB ICD improves persistence.^29,37–39^ In evaluating the contributions made by the structural domains to the function and efficacy of the CAR-T cells, parity in the design was paramount to revealing the contribution made by either the CD8HTM or CD28HTM CAR, leading us to include both the CD28 and 4-1BB co-stimulatory domains in the design. Moreover, in the context of controlling solid tumors, the 28BBζ design has demonstrated *in vivo* efficacy in controlling bulky metastases as well as GUCY2C-expressing tumors.^19,40^ In clinical trials using a 28BBζ design directed toward CD19, these CARs have been effective and safe; however, there is a shortage of true comparative studies between second- and third-generation designs utilized clinically.^41,42^ We also opted for this study to treat the hinge and transmembrane domains as a structural unit, as is the current clinical practice utilizing the hinge and transmembrane domain from either CD8ɑ or CD28.

As we expected, the two CARs did not demonstrate any variability in the final product of the CAR-T cells at the end of the culture period in terms of expansion or viability. In fact, in all CAR-independent T-cell functions, including memory and exhaustion phenotypes, there were no observed differences. In the manufactured product, we did observe a statistically significant difference in both the transduction of CD8^+^ and CD4^+^ T cells and in the percentage of GFP^+^ T cells between the two CARs. The higher transduction efficiency observed in CD4^+^ T cells has been reported in the literature before^24,25,43^; however, a mechanism has not yet been elucidated. We speculate that this difference is specific to vesicular stomatitis virus glycoprotein (VSV-G) pseudotyped lentivirus. In 2013, the low-density lipoprotein receptor (LDL-R) was identified as the host cell receptor for VSV-G viral entry. RNAseq data from The Human Protein Atlas (proteinatlas.org) demonstrates that resting CD8^+^ T cells have 4.2 LDL-R transcripts per million (TPM) and CD4^+^ T cells have 6.2 LDL-R TPM. When T cells are activated, LDL-R expression is upregulated in both CD8^+^ and CD4^+^ T cells, producing 86.2 and 112.6 TPM, respectively.^44^ The increased transcript level produced by the activated CD4^+^ T cells relative to the activated CD8^+^ T cells presents the most plausible explanation for the difference in transduction efficiency. The low level of LDL-R on resting T cells also explains why T cells require activation for efficient transduction. In fact, Natural Killer (NK) cells, which are more resistant to lentiviral transduction than primary human T cells, show improved transduction after treatment with rosuvastatin for the express purpose of increasing LDL-R cell surface expression and promoting improved viral entry.^45^ With respect to the CD28HTM CAR having a higher percentage of GFP^+^ T cells compared with the CD8HTM CAR for both the CD8^+^ and CD4^+^ CAR-T cells, this difference is most likely due to variability in the quantification of viral titers as well as the direct administration of the lentivirus to the cells. When comparing the GFP MFI of the CD8HTM and CD28HTM CAR-T cells, which is a reflection of the per-cell fluorescence intensity, the two CARs are equivalent. While there are more total cells present that express GFP within a given subset, there is no indication that an individual cell is being infected with more viral particles and expressing more GFP as a result. Moreover, in direct functional comparisons between CD8HTM and CD28HTM, populations were balanced using donor-matched untransduced cells, so that the number of CAR^+^ and total T cells were equivalent between the groups both *in vitro* and *in vivo*.

What was not expected was the difference in antigen affinity between the two CARs, considering that the scFv used in both CARs was the same. It has been demonstrated that hinge length can have an impact on efficacy; however, these differences are believed to be due to the ideal cell-to-cell distance of 15 nm between the T cell and target cell.^46,47^ In this assay, the antigen is soluble, suggesting that differences other than hinge length are responsible. Biophysical characterization by Chen et. al. of the CD8ɑ hinge demonstrated that CAR-T cells expressing a CD8ɑ hinge were superior killers *in vitro* compared with a CD28 hinge-expressing CAR when killing low antigen-expressing target cells, but not when antigen levels were high, which is what we observed with the CD8HTM CAR killing the LoVo cells.^48^ They propose that increased flexibility makes it more favorable to achieve this ideal intermembrane distance; however, this flexibility may also have benefits within the CAR molecule itself, improving the efficiency of folding of the scFv, conferring a slight but significant increase in affinity that is revealed when antigen density is low and affinity-stabilized interactions promote a complementary increase in avidity, allowing for the formation of a more productive T-cell synapse. A slight change in the folding of the scFv due to the intrinsically more flexible CD8ɑ hinge may also explain why the protein L binding is significantly higher in the CD4^+^ T cells, between the CD8HTM and CD28HTM CAR, but not observed with G4S detection. Slight conformational differences in the scFv may also have an impact on the affinity between protein L and the light chain framework. Coupled with higher CAR surface expression compared to the CD8^+^ T cells, slight affinity changes would be more apparent in binding assays within the CD4^+^ T-cell population, which we observed.

In many respects, the two CARs produce T cells that function similarly. While the CD8HTM CAR produced a quantitatively significant increase in inflammatory cytokines, the qualitative abundance of the respective cytokines is similar, suggesting that the signaling directed through the CAR is being amplified rather than diversified, i.e. previously silenced signaling pathways are not being activated by the CD8HTM CAR compared to the CD28HTM CAR. Nevertheless, this difference in cytokine abundance still has functional consequences. The reduction in affinity, which leads to a reduction in total granzyme B and cytokine production, may explain why the killing observed *in vitro* is less complete with the CD28HTM CAR in low antigen conditions. Antigen expression being a Gaussian continuum, one would anticipate that cells from the same cell line with stochastically higher antigen levels would be killed more rapidly in the *in vitro* assay, while lower expressing cells would take longer, creating a selection pressure promoting the survival of the lowest antigen-expressing cells. The assumption is that every target cell that is killed is killed directly by the CAR-T cell, but inflammatory cytokines can also activate pro-apoptotic pathways within the target cells. Inflammatory bowel diseases occur because the intestinal epithelium undergoes apoptosis in response to inflammatory cytokines, not by perforin- and granzyme-mediated cytolysis.^49^ The increased affinity of the CD8HTM CAR allows for the maintenance and sustainability of the anti-tumor response, not solely because of the direct, synaptic interaction, but also because of the inflammatory milieu in which these T cells function. This translates to the difference we see *in vivo*, where the balance of selective pressure is reversed. Target cells *in vitro* are given 24 hours to establish a microenvironment, whereas target cells in an animal are given two weeks. *In vivo*, the T cells are more susceptible to immunosuppressive effectors, so even though antigen is no longer a limiting factor, with the T84 model, the higher affinity, improved sensitivity, increased signaling, and subsequent response with the CD8HTM CAR-T cells has a cumulative effect that results in superior anti-tumor efficacy *in vivo*.

The compartmentalized, modular approach to CAR design often disregards the holistic ramifications that changing a single component may have; and demonstrates that the reliance on empiricism in the field of CAR-T cells is still, as yet, unrelenting. What has previously been presented as being wholly attributable to ICDs or scFvs may not be as straightforward as previously thought and suggests that the myopic definition of efficacy be expanded into a more formalized rubric for assessing CARs and CAR design within the field. There are still many unknowns with the CD8HTM and CD28HTM CARs, including the kinetics of synapse formation, how the synapse is ordered, if there are differences in proximal signaling, how receptor endocytosis and recycling compare, what signals are promoting the difference in granzyme B production, and how are these CAR-T cells behaving within the tumor. Despite these important, unanswered questions, this study has allowed us to identify a clinical candidate for the treatment of metastatic CRC. Although CAR-T cell therapy has not yet witnessed the successes in solid tumors as have been experienced in hematological malignancies, these iterative increases in our understanding of how design impacts the CAR, the T cell, and the target, will eventually allow for CAR-T cells to be successful in the solid tumor space.

## Methods

### Production of lentiviral vectors

Cassettes for CAR transfer plasmids were synthesized and cloned (GenScript Biotech, Piscataway, NJ) into the pCDH-EF1ɑ-MCS-T2A-GFP lentiviral transfer vector (CD525A–1, System Biosciences) using the *XbaI* and *BamHI* restriction enzymes. All plasmids, including lentiviral packaging and envelope plasmids, were transformed into NEB Stable Competent E. coli (C3040H, New England Biolabs). E. coli were cultured in LB Broth, Miller (BP1426–2, Fisher Scientific) supplemented with 1.9% Bacto Yeast Extract (212750, Thermo Fisher Scientific) and 100 ug/mL Ampicillin (A8351-5 G, Sigma-Aldrich). DNA was purified from overnight bacterial cultures using the Purelink Expi Endotoxin-Free Maxi Plasmid Purification Kit (A31231, Thermo Fisher Scientific). DNA pellets were resuspended in Endotoxin-free water to a final concentration of ~ 1 ug/uL. Plasmid DNA was stored at −20°C.

### High titer lentivirus production

T-225 flasks were coated with 5 ug/cm^2^ poly-d-lysine (354210, Corning), washed with DPBS (21–031-CV, Corning), and allowed to dry before 28.4 million HEK293T/17 cells (CRL11268, American Type Culture Collection (ATCC)) were seeded in cell culture medium consisting of Advanced DMEM (12491023, Thermo Fisher Scientific) supplemented with 5% heat-inactivated FBS (A38400–01, Gibco) and 1X GlutaMAX (35050–061, Gibco). The next day, Lipofectamine 3000 transfection reagent (L3000150, Thermo Fisher Scientific) was used to deliver the lentiviral plasmids in the following amounts: 17.7 ug CAR transfer plasmid, 14.7 ug pRSV-Rev (12253, Addgene), 31 ug pMDLg/pRRE (12251, Addgene), and 7.6 ug pMD2.G (12259, Addgene) in Advanced DMEM (no supplementation). Six hours post-transfection a complete media exchange was performed. Media was collected at 24- and 52-hours post-transfection and filtered using a 0.45 um ɑPES filter unit (09–740-63E, Fisher Scientific). Lentivirus was concentrated using a 4X Polyethylene Glycol 8000 (BP233–1, Fisher Scientific) solution that was incubated on a wave rotator at 4°C overnight followed by centrifugation at 1600×g for 1 hour at 4°C. Lentiviral pellets were resuspended at a 200X concentration in lentivirus storage buffer: 10 mM Tris, pH 7.4 (648315-100 ML, EMD Millipore), 10% lactose (61339-25 G, Sigma-Aldrich), 25 mM Proline (81709-10 G, Sigma-Aldrich) in DPBS. Virus was stored at −80°C. Lentivirus titer was determined by transducing HEK293T/17 cells in the presence of 0.8 ug/mL Polybrene (TR-1003-G, Millipore Sigma), measuring the percentage of GFP^+^ cells using a BD FACSymphony A5 SORP Flow Cytometer (BD Biosciences).

### CAR-T cell production

Human T cells were isolated from the peripheral blood mononuclear cells (PBMCs) of six healthy donor leukopaks (200-0092, Stemcell Technologies) using magnetically sorted negative selection (130-096-535, Miltenyi Biotec). T cell culture medium was composed of RPMI-1640 (10-041-CV, Corning) supplemented with 10% heat-inactivated FBS (A38400–01, Gibco), 1X Insulin-Transferrin-Selenium (ITS-G 41,400–045, Gibco), 10 mM N-Acetyl-L-cysteine (A9165, Millipore Sigma), 1X GlutaMAX (35050–061, Gibco), 1X Glucose solution (A24940–01, Gibco), 1X Sodium Pyruvate (11360–070, Gibco), 1X MEM Non-Essential Amino Acids (11140–050, Gibco), 1X HEPES Buffer (15630–080, Gibco), 1X Penicillin-Streptomycin (15140–122, Gibco), and 55 uM 2-Mercaptoethanol (21985–023, Gibco). At the start of culture, T cells were seeded into conventional 6-well plates (3516, Corning) at a density of 1 million cells/mL in 4 mL of culture medium. T cells were activated using CD3/CD28/CD2 magnetic beads (1:1 bead-to-cell ratio, 130-091-441, Miltenyi Biotec) in the presence of 10 ng/mL human IL-7 and 10 ng/mL human IL-15 (BRB Preclinical Biologics Repository, NCI Biological Resources Branch, Frederick, MD). T cells were transduced with CAR LV at an MOI of 5 with 0.8 ug/mL Polybrene (TR-1003-G, Millipore Sigma) 24 hours after activation. Activation beads were magnetically removed 72 hours after activation and the T cells were transferred to 6-well G-Rex plates (80240 M, Wilson Wolf). T cells received fresh culture medium supplemented with IL-7 and IL-15. Cytokines were replenished every three days. A media exchange occurred on Day 9 post-activation, and cells were collected on Day 12. T-cell concentration and viability were assessed using the Guava MUSE Cell Analyzer (Cytek Biosciences). T cells were cryopreserved using CryoStor CS10 (07930, Stemcell Technologies) at a density of 20 million cells/mL. Prior to experiments, CAR-T cells were thawed in RPMI-1640 (no supplementation) warmed to 37°C, diluted to 1 million cells/mL in T-cell culture medium with IL-7 and IL-15, and allowed to recover in the cell culture incubator for 3 ± 1 days.

### Flow cytometric evaluation of CAR T-cells

For all flow cytometry experiments, Fc receptors were blocked using Human TruStain FcX Fc Receptor Blocking Solution (422302, BioLegend). All fluorophore-conjugated antibody cocktails contained 10% Brilliant Stain Buffer Plus (566385, BD Biosciences). Triplicate samples were always run for every test sample. The BD FACSymphony A5 SORP was used for all flow cytometry experiments. For each sample 30,000 live, single cell gated events were collected. All analysis was done using FlowJo, v10 software (BD Biosciences).

To determine the level of CAR surface expression, Protein L or the anti-G4S linker antibody were used as detection methods. CAR-T cells were incubated with 1 ug/mL recombinant His-tagged protein L (RPL-P3141, ACRO Biosystems) for 45 minutes at 4°C. Cells were incubated with fluorophore-conjugated antibodies for 30 minutes at 4°C (BV421-CD4, Clone RPA-T4, 300532, BioLegend; BB700-CD8, Clone RPA-T8, 566452, BD Biosciences, PE-G4S linker, Clone E720V, Cell Signaling Technology; Alexa Fluor 647-Penta-His 35,370, Qiagen). Live cells were identified using SYTOX AADvanced Dead Cell Stain Kit (S10274, Thermo Fisher Scientific). For cell surface quantification, BD Quantibrite PE beads (340495, BD Biosciences) were used to generate a standard curve in which the PE-G4S geometric mean values were used to determine the number of receptors per cell.

GUCY2C binding was performed using recombinant, extracellular domain GUCY2C protein that encoded the 23 amino acid (aa) GUCY2C signal peptide, an N-terminal 6× His affinity tag, the 408aa GUCY2C extracellular domain and a C-terminal 8aa Strep II tag. Protein was synthesized and purified by GenScript Biotech (Piscataway, NJ). Protein concentration was determined using Pierce BCA Protein Assay Kit (23227, Thermo Fisher Scientific) and SDS-PAGE and Western blot analysis (performed by GenScript) determined the molecular weight to be 70 kDa. To cover the entire logarithmic dilution range, 10-fold dilutions were established starting at either 1000 nM or 300 nM in FACS buffer: 1% Bovine Serum Albumin (BP1600–100, Fisher Scientific), 0.1% Sodium Azide (AC190381000, Fisher Scientific) in DPBS. CAR-T cells were incubated with dilutions of GUCY2C protein for 1 hour at 4°C. T cells were then incubated with the antibody cocktail (BUV805-CD3, Clone OKT3, 750970, BD Biosciences; BV BV421-CD4, Clone RPA-T4, 300532, BioLegend; BB700-CD8, Clone RPA-T8, 566452, BD Biosciences, Alexa Fluor 647-Penta-His 35,370, Qiagen). Immediately upon the conclusion of staining, the cells were placed on ice and immediately analyzed on the flow cytometer. Antigen binding curves were generated by normalizing GFP^+^ CD4^+^ or GFP^+^ CD8^+^ MFIs to the highest and lowest values within a sample set.

Intracellular cytokine (ICS) staining was performed by coating 24-well tissue culture plates with 10 ug/mL GUCY2C extracellular domain protein or bovine serum albumin as a negative control. 1 million CAR-T cells were added per well with 1X protein transport inhibitor (00-4980-03, Thermo Fisher Scientific). PMA/Ionomycin was added to positive control wells for stimulation (Cell Stimulation Cocktail, 00-4970-03, Thermo Fisher Scientific). To identify CAR^+^ T cells, Alexa Fluor 488-G4S antibody (50515 L, Cell Signaling Technology) was added at a final dilution of 1:800 to each well. Cells were incubated in the presence of antigen for 6 hours in a cell culture incubator, at which point the cells began the staining procedure. Fixable Viability Stain (FVS) 575 V (565694, BD Biosciences) was used to identify the viable cell population. Cell surface antigens were stained at 4°C for 30 minutes (BUV395-CD45, Clone HI30, 363-0459-42, Thermo Fisher Scientific; BUV805-CD3, Clone OKT3, 750970, BD Biosciences; BUV563-CD4, Clone RPA-T4, 741353, BD Biosciences; BB700-CD8, Clone RPA-T8, 566452, BD Biosciences). Cells were fixed with 4% paraformaldehyde (PFA) in PBS (J19943.K2, Thermo Fisher Scientific) for 20 minutes at 4°C. Cells were stored at 4°C overnight before proceeding with intracellular staining. Cells were permeabilized for 15 minutes at room temperature using Perm/Wash buffer (554723, BD Biosciences) diluted in water. Antibodies for intracellular cytokine staining were diluted in perm/wash solution. The following antibodies were used: BUV737-IL2, Clone MQ1-17H12, 612836, BD Biosciences; BV421-IFNɣ, Clone 4S.B3, 564791, BD Biosciences; RB780-granzyme B, Clone GB11, 568705, BD Biosciences; Alexa Fluor 647-TNFɑ, Clone MAb11, 502916, BioLegend. Staining was performed for 30 minutes at 4°C. A final 2% PFA fixation was performed. Cells were analyzed on the flow cytometer within 24 hours of completing the staining protocol.

Flow cytometry for T-cell phenotypic markers consisted of the FVS525V, followed by the following surface markers: BUV395-CD45, Clone HI30, 363-0459-42, Thermo Fisher Scientific; BUV805-CD3, Clone OKT3, 750970, BD Biosciences; BUV563-CD4, Clone RPA-T4, 741353, BD Biosciences; BB700-CD8, Clone RPA-T8, 566452, BD Biosciences; Alexa Fluor 488-G4S, Clone E720V 50,515 L, Cell Signaling Technology. For memory phenotyping the following markers were used: BV421-CCR7, Clone 2-L1-A 566,743, BD Biosciences; Alexa Fluor 647-CD45R, Clone HI100, 304112, BioLegend. For exhaustion marker analysis, the following antibodies were used: BUV661-CD39, Clone TU66, 569788, BD Biosciences; BV421-PD-1, Clone MIH4, 564323, BD Biosciences; PE-CF594-Lag-3, Clone T47–530, 565718, BD Biosciences; Alexa Fluor 647-Tim-3, Clone 7D3, 565558, BD Biosciences. Antibody staining was performed for 30 minutes at 4°C. Cells were fixed with 4% PFA at 4°C for 20 minutes. Cells were stored at 4°C until analysis on the flow cytometer.

### GUCY2C mRNA analysis

SW620 (CCL227, ATCC), LoVo (CCL229, ATCC), LS174T (CL-188, ATCC), and T84 (CCL-248, ATCC) cell lines were plated in 24-well tissue culture plates. Using an RNeasy kit (74104, Qiagen), samples were lysed in RLT buffer supplemented with 550 uM 2-mercaptoethanol. RNA was purified on spin columns according to manufacturer instructions. RNA concentrations and purity were measured with a Nanodrop 1000 (Thermo Fisher Scientific). RNA was reverse transcribed to complementary DNA (cDNA) using the TaqMan Reverse Transcription kit according to the manufacturer’s instructions (N8080234, Thermo Fisher Scientific). Transcripts were quantified by qRT-PCR using Taqman primer probes (Human GUCY2C Assay ID Hs00990106_m1, Thermo Fisher Scientific; Human β-Actin Assay ID Hs01060665_g1, Thermo Fisher Scientific) on a QuantStudio™ 3 Real-Time PCR System (Thermo Fisher Scientific), with TaqMan Universal PCR Master Mix (4318157, Thermo Fisher Scientific) according to instructions.

### GUCY2C Western blot analysis

CRC cell lines were cultured in 10 cm dishes. Radio-Immunoprecipitation Assay (RIPA) buffer was supplemented with 1X HALT Protease and Phosphatase Inhibitor cocktail, EDTA-free (78441, Thermo Fisher Scientific) and 1 mM Phenylmethylsulfonyl fluoride (PMSF, 36978, Thermo Scientific) and the solution was placed on ice. Culture plates were washed in ice-cold DPBS three times, followed by 1 mL of RIPA buffer solution. Cells were scraped, and the contents were transferred to microcentrifuge tubes. Cells were placed on a tube rotator at 4°C for 30 minutes. Tubes were centrifuged at 12,000 RPM for 10 minutes. Supernatants were transferred to ice-cold microcentrifuge tubes and immediately placed in −20°C storage.

Protein concentration was measured using the Pierce BCA Protein Assay Kit (same as above). Samples were diluted with 4X Invitrogen NuPage LDS Sample Buffer (NP0007, Fisher Scientific) and Invitrogen Novex NuPage Sample Reducing Agent (NP0009, Thermo Scientific). Samples were boiled for 10 minutes at 90°C and immediately placed on ice. Samples were loaded into NuPage 4–12% Bis-Tris Protein Gels (NP0336BOX, Thermo Fisher Scientific) along with Invitrogen Novex Sharp Pre-Stained Protein Ladder (LC5800, Thermo Fisher Scientific). Gels were run at 130 V for 90 minutes. Gels were transferred to the iBlot 3 Transfer Stack, nitrocellulose (IB33002X3, Thermo Scientific) using a seven-minute transfer program. The membrane was blocked in 10% nonfat dry milk (M0841, LabScientific) in PBS with Tween-20 (PBS-T). Membranes were probed using a Rabbit anti-human GUCY2C antibody (37517, Cell Signaling Technology) or Rabbit anti-human β-Actin (8457, Cell Signaling Technology). Both antibodies were used at a dilution of 1:1,000. An HRP-conjugated Goat anti-Rabbit IgG secondary antibody was used at 1:12,500 in PBS-T. Thermo Scientific SuperSignal West Femto Chemiluminescent Substrate (34096, Thermo Fisher Scientific) was used to detect the bands on a ChemiDoc MP Image System (Bio-Rad Laboratories, Inc.). Images were analyzed using Image Lab, version 6.1.0 (Bio-Rad Laboratories, Inc.).

### In vitro cytotoxicity assay

LoVo cells were cultured in F-12K medium (10–092-CV) supplemented with 10% FBS (35–010-CV, Corning). LS174T cells were cultured in Eagle’s Minimal Essential Medium (10–010-CV, Corning) supplemented with 10% fetal bovine serum (35–010-CV, Corning), 1X GlutaMAX (35050–061, Gibco), 1X NEAA (11140–050, Gibco), and 1X Sodium Pyruvate (11360–070, Gibco). T84 cells were cultured in Advanced DMEM/F-12 50%/50% (12634028, Thermo Fisher Scientific) supplemented with 5% FBS (35–010-CV, Corning) and 1X GlutaMAX (35050–061, Gibco). When the cells were ready to be used in cytotoxicity experiments, the cells were trypsinized (25–053-CI, Corning), filtered through a 30 um sterile MACS SmartStrainer (130-098-458, Miltenyi Biotec), and counted using a hemocytometer. Cells were resuspended to a density of 8.0 × 10^5^ cells/mL in the respective growth media for each cell line.

Cytotoxicity was measured in real-time using the Agilent xCELLigence Real Time Cell Analysis (RTCA) SP – Single Plate analyzer (Agilent Technologies, Inc.). 100 uL of each cell line’s respective media was added to the well of an E-Plate 96 (5232368001, Agilent Technologies, Inc.) to establish a baseline impedance measurement for each well. 4.0 × 10^4^ CRC cell line target cells were added to each well in the cell culture hood. The plate was allowed to incubate for 30 minutes at room temperature before being placed into the xCELLigence RTCA machine (located within a cell culture incubator set to 37°C and supplied with 5% CO_2_). The cells were allowed to adhere for 24 hours and only when the cell index (an arbitrary unit measurement of current impedance) was above 1.0 were cells used in cytotoxicity experiments. CAR-T cells were balanced in terms of GFP^+^ CAR-T cells as well as total T cells. T cells were collected, counted, and resuspended so that the CAR^+^ populations were at a density of 1.6 × 10^6^ cells/mL. T cells were resuspended in RPMI-1640 (10-041-CV, Corning) without any supplementation or cytokines. 8.0 × 10^4^ CAR-T cells in 50 uL were added to each well for an E:T ratio of 2:1. The E-Plate was placed in the xCELLigence RTCA machine and cell impedance measurements were collected every 15 minutes. The RTCA Software Pro (Agilent Technologies, Inc.), which is used to run the machine, was also used to analyze the data.

### In vivo mouse tumor study

Animal experiments were conducted in accordance with an IACUC approved animal protocol and followed the guidelines for use of research animals stipulated by Thomas Jefferson University. A lentiviral construct was made using a modified pCDH-EF1ɑ-MCS-T2A-GFP backbone where the GFP reporter was replaced with the fluorescent mCherry protein. The Click Beetle Red luciferase (CBRluc) was cloned into the multiple cloning sites using *XbaI* and *BamHI* restriction enzymes to generate the pCDH-EF1ɑ-CBRluc-T2A-mCherry lentiviral vector. DNA was transformed, amplified, and purified and lentivirus was generated as stated above. T84 cells were transduced with concentrated lentivirus at an MOI of 1 in the presence of 0.8 ug/mL polybrene. Cells were expanded and then flow-sorted on live singlets for 50% of the mCherry population surrounding the median mCherry fluorescent signal to isolate a pure, uniform population of transduced cells. Flow sorting was performed using a BD FACSMelody sorter. Flow-sorted T84-CBRluc-T2A-mCherry (T84-Luciferase) cells were expanded in tissue culture and cryopreserved in Fetal Bovine Serum (FBS, 35–010-CV, Corning) with 10% DMSO (PI20688, Fisher Scientific). Prior to administration, T84-Luciferase cells were removed from liquid nitrogen storage and cultured using Advanced DMEM/F-12 50%/50% (12634028, Thermo Fisher Scientific) supplemented with 5% FBS (35–010-CV, Corning) and 1X GlutaMAX (35050–061, Gibco). The cells were cultured and expanded for 10 days before being trypsinized (25–053-CI, Corning) and filtered through a 30 um sterile MACS SmartStrainer (130-098-458, Miltenyi Biotec). Cells were counted and resuspended in cold DPBS at a final concentration of 12.5 × 10^6^ cells/mL. Cells were kept on ice for injection.

Four-week-old female NSG-MHC I/II DKO mutant mice (025216, The Jackson Laboratory) were injected intraperitoneally with 2.5 × 10^6^ T84-Luciferase cells in 200 uL using a 0.5 mL tuberculin syringe (14-826-79, Fisher Scientific). On the day of tumor cell implantation, a culture of CAR-T cells using a single donor was started following the above methods for CAR-T cell production. On Day 12, CAR-T cells were evaluated for transduction efficiency using flow cytometry to determine the percentage of GFP^+^ in the CD8HTM and CD28HTM CAR-T cell populations. On Day 13, animals were imaged using an IVIS Spectrum In Vivo Imaging System with a five-mouse manifold (Perkin Elmer). The luminescence signal in Photons/second/centimeter^2^/steradian was used to rank all of the animals in terms of luminescence signal. Four mice surrounding the median of the ranked luminescence intensities were allocated to the untreated group to control for unimpeded tumor growth. The remaining animals were allocated using block randomization to the CD8HTM or CD28HTM group. Fourteen days after tumor implantation, CAR-T cells were collected and counted. The two CAR-T cell groups were balanced in terms of the percent of GFP^+^ cells as well as total T cells, using donor-matched untransduced T cells. Thus, all animals received the same number of CAR-T cells in the same number of total T cells. 3.0 × 10^6^ CAR^+^ T cells were injected intravenously via the tail vein.

Starting seven days post tumor implantation, animals began undergoing *in vivo* bioluminescent imaging. Animals were anesthetized using inhaled isoflurane. Mice were sedated for three minutes, and then each animal was injected subcutaneously with 250 uL of a 15 mg/mL solution of D-Luciferin, potassium salt (LUCK-4 G, Gold Biotechnology) dissolved in DPBS (21–030-CV, Corning). Animals were returned to the isoflurane anesthesia chamber. Five minutes after the administration of D-Luciferin solution, animals were imaged in the IVIS using a 10-second exposure. Throughout the course of the experiment, animals that were responsible for saturating the optical system beyond the linear range of the photon detector would be removed after imaging, and the remaining animals would be re-imaged to provide a more accurate reading of the luminescent signal. Animals were imaged bi-weekly for a 6-week period following CAR-T cell treatment. Images were analyzed using Aura In Vivo Imaging Software (Spectral Instruments Imaging). All images were set to the same color range minimum, color range maximum, and color range threshold. To determine the baseline luminescence for each day of imaging, a single cage of five animals received vehicle (DPBS) alone on the day of tumor implantation. On Day 14, these animals received the same number of untransduced total T-cells as the CAR-T-treated groups. These animals were imaged as above throughout the experiment.

## Data Availability

The data that support the findings of this study are available from the corresponding author, AES, upon reasonable request. All figures presenting data generated in this study, including all statistical analyses, were performed using GraphPad Prism, version 10.2.3. Comparisons employed T-test, One-way ANOVA, Two-way ANOVA, and Kaplan – Meier two-sided log-rank tests as appropriate.
